# Death from Chloroform

**Published:** 1875-04

**Authors:** 


### DEATH FROM CHLOROFORM.

At Boston, on Saturday, October 3d, in the case of Linscott who died in a dentist's chair, from the inhalation of chloroform, the coroner's jury declared themselves of the opinion that, with our present knowledge of chloroform, its use as an anæsthetic is wholly unjustifiable, and they recommended the passage of a law forbidding its administration.

